# Coronary artery disease epidemic in Pakistan: more electrocardiographic evidence of ischaemia in women than in men

**DOI:** 10.1136/hrt.2007.120774

**Published:** 2007-07-23

**Authors:** T H Jafar, Z Qadri, N Chaturvedi

**Affiliations:** 1 Clinical Epidemiology Unit, Department of Community Health Sciences, Aga Khan University, Karachi, Pakistan; 2 International Centre for Circulatory Health, National Heart and Lung Institute, Imperial College London, UK

## Abstract

Indo-Pakistani populations have one of the highest risks of coronary artery disease (CAD) in the world. A population-based, cross-sectional survey was conducted on 3143 adults aged ⩾40 years from 12 randomly selected communities in Karachi, Pakistan. Apart from smoking, women had more CAD risk factors (diabetes, hypertension, obesity, dyslipidaemia) than men. Definite CAD (history and Q waves on ECG) was more prevalent in men than in women (6.1% vs 4.0%; p = 0.009). In contrast, ischaemic and major ECG changes were twice as prevalent in women as in men (29.4% vs 15.6%, and 21.0% vs 10.5%; p<0.001 for each, respectively). All measures of CAD were strongly predicted by the metabolic syndrome, but that failed to account for the greater prevalence of ECG abnormalities in women than in men. The findings indicate that one in five middle-aged adults in urban Pakistan may have underlying CAD. Women are at greater risk than men.

**Trial registration number:** NCT00327574

People of Indo-Asian origin have one of the highest susceptibilities to coronary artery disease (CAD) in the world,^1^ ^2^ and it is therefore unsurprising that CAD is now the leading cause of death in the Indo-Pakistan subcontinent.^3^

Meta-analysis of previously conducted studies suggests an almost 2.5-fold rise in the prevalence of CAD in two decades—from 3.6% in the 1970s to 9.5% in the 1990s in people aged ⩾35 years in urban India.^4^ With increasing urbanisation in the subcontinent, CAD prevalence can only continue to rise, and this underlines the need to understand risks and determinants of disease in this population to inform preventive strategies.

We and others have reported that unlike European populations, the burden of cardiovascular risk factors in Indo-Asians, such as hypertension, diabetes, dyslipidaemia, obesity, including central obesity, and physical inactivity, are at least equivalent if not greater in women than in men.^5^^–^8 Correspondingly, the gender difference in prevalent CAD may also be attenuated, or even abolished in Indo-Asians, depending upon the diagnostic criteria employed.^6^^–^9 Population-based studies require unbiased, non-invasive measures of CAD. In developing countries, in particular, doctors’ reports may be biased by gender differences in healthcare seeking behaviour, referral and diagnostic preferences. On the other hand, ECG criteria are thought to be of less prognostic significance in women.^10^ ^11^ However, gender differences in prevalent CAD according to available non-invasive criteria for diagnosis, and their associations with risk factors, have not been formally studied in large population samples from the Indo-Pakistan subcontinent, making the choice of definition difficult.

We therefore conducted this study to determine gender differences in the prevalence of CAD using non-invasive criteria, and their association with cardiovascular risk factors in a population sample from Karachi, Pakistan.

## METHODS

Ethical approval was obtained from ethics review committee at the Aga Khan University, Pakistan.

### Study design

The study design comprised cross-sectional analysis of a population-based sample studied for recruitment to a cluster randomised trial of strategies to control hypertension. (Trial registration number NCT00327574.) All subjects aged ⩾40 years and able to give their consent were included in the study. Poorly mobile patients, those mentally incompetent and unable to give consent, or patients who had known advanced liver or kidney failure or pregnancy were excluded.

### Sampling details

The Federal Bureau of Statistics has divided the city of Karachi into 5000 clusters each of about 250 households. On average, each household contains seven people, 20% of whom are aged ⩾40 years. About 85% of all clusters (4200) are in the low-income group neighbourhoods (average household income less than $120/month) and were mapped into 12 geographically contiguous areas of 350 clusters each. One cluster was randomly selected (computer generated) from each area for inclusion in the study. A census was done, and a listing of all people from all households in the selected areas was made.

Trained community health workers paid home visits to the selected subjects and invited them to participate in the study. All subjects were evaluated by a rigorously trained field team (comprising field workers, nurses and technicians) after obtaining informed consent. The evaluation included the following: (*a*) history and physical examination; (*b*) questionnaire detailing smoking status, food frequency questionnaire and other lifestyle factors, self-reported doctor diagnosis of heart disease, the World Health Organisation Rose questionnaire^12^ for angina, and the international physical activity questionnaire; (*c*) assessments of blood pressure with a calibrated automated device (Omron HEM-737 blood pressure monitor, Omron Healthcare Inc, Illinois, USA) in the sitting position after at least 20 minutes of rest; (*d*) anthropometry (height, weight, waist and hip circumferences); (*e*) laboratory tests for risk factors for cardiovascular disease (CVD) including fasting blood glucose (Synchron Cx-7/Delta, Beckman, USA), lipid profile (HITACHI-912, Roche Japan), spot urine microalbumin ((reagent: Pyrogallol Red plus Sodium molybdate, Synchron Cx-7/Delta) Array Systems, Beckman) and urine creatinine (Synchron Cx-7/Delta); (*f*) and a 12-lead electrocardiogram (ECG) coded to the Minnesota code by a grader in the UK who was unaware of all information except the participant identity number. All measurements were performed to a standard protocol that conforms to the international standards for definitions and measurements. The study was conducted over 1 year (2004–5).

### Statistical analysis

Definitions of CAD used a combination of criteria: definite CAD (defined as a doctor’s report of a heart attack or Q waves on ECG (Minnesota codes 1-1 to 1-2)), positive response to Rose angina questionnaire, ischaemic ECG (Minnesota codes 1-3 or 4-1 or 4-2 or 4-3 or 5-1 or 5-2 or 5-3 or 7-1), and major ECG abnormalities (ECG Minnesota codes: 4-1 or 4-2 or 5-1 or 5-2 or 6-1 or 6-2 or 7-1 or 7-2 or 8-1 or 8-3.^13^ ^14^)

Hypertension was defined as a mean systolic pressure ⩾140 mm Hg or a mean diastolic pressure ⩾90 mm Hg measured 20 minutes apart, on two separate occasions, or taking antihypertensive drugs.

Diabetes was defined as fasting blood sugar ⩾7.0 mmol/l, or taking antidiabetic drugs.^15^

Overweight and obesity was defined as body mass index of ⩾23 kg/m^2^.^16^

Central obesity was defined as a waist circumference (measured at the mid-point between the iliac crest and the lower rib margin) of ⩾80 cm in women and ⩾90 cm in men.^17^

Metabolic syndrome was defined as the concomitant presence of at least three of the following: hypertension, high triglycerides (>1.70 mmol/l); or low HDL cholesterol (<1.04 mmol/l in men or <1.30 mmol/l in women), impaired glucose tolerance (fasting glucose ⩾7.0 mmol/l), or central obesity. The definition was based on the components included in the third report of the National Cholesterol Education Program (NCEP) guidelines with the modification for definition of central obesity for South Asian populations, and levels of blood pressure based on the Joint National Committee (JNC) VII for diagnosis of hypertension.^18^

Physical activity was defined based on metabolic equivalent (METS) per week collected from the international physical activity questionnaire.^19^

Low level of education was defined as less than 7th grade of formal school education.

Economic status was measured by monthly income and by ownership of household items.

Past smokers were defined as those who smoked ⩾100 cigarettes in their life time, but were not currently smoking.

All data analyses were performed in SAS version 9.1.3 (SAS Institute Inc, Cary, NC, USA). The prevalence of outcomes (95% CI) was calculated for the studied population, stratified by sex, after accounting for cluster sampling. Associations between risk factors and measures of CAD were sought, again stratified by sex, and multivariable models were built to assess the independence of such associations. We also combined the sexes to explore risk factors which might account for gender differences in CAD outcomes. We performed logistic regression analysis specific for complex survey designs which accounted for cluster sampling for the outcomes using various definitions of CAD: (*a*) definite CAD; (*b*) ischaemic ECG changes; and (*c*) major ECG abnormalities. For each model, those without the primary outcome and with other definitions of CAD or a positive response to the Rose angina questionnaire were excluded from the analysis. This rationale was used to generate a comparator group “clean” of possible CAD. Candidate predictors for these models included sociodemographic variables such as age, gender, level of education, diet, physical activity, economic status, smoking status, individual biological risk factors and the composite measure of the metabolic syndrome. A forward selection method was used with entry criteria of p<0.1 for factors associated with CAD, and those with p<0.05 were retained in the final model.

## RESULTS

A target sample population of 3546 subjects aged ⩾40 years, stratified by sex were invited to enrol in the study; 3143 (88.6%) consented. The mean (SD) age of participants was 51.6 (10.8) years and 1503 (48%) were men. Table 1 shows the characteristics of the study population. Women were more likely to have diabetes, hypertension, and raised low-density lipoprotein cholesterol levels, more likely to be obese, and were much less likely to be physically active than men. They were, however, less likely to be current or ex-smokers, and had lower triglyceride levels. The prevalence of the metabolic syndrome in women was substantially greater than in men (51.2% vs 32.4%, p<0.001).

**Table 1:** Characteristics of men and women in mid- to low-income communities in Karachi

Variables	All (n = 3143) No (%)	Men (n = 1503) No (%)	Women (n = 1640) No (%)	p Value
Age (years), mean (SD)	51.6 (10.8)	51.9 (10.6)	51.3 (11.0)	0.147
Formal schooling status (%)				
Middle school (grade 7 or less)	2137 (68.0)	881 (58.6)	1256 (76.6)	<0.001
Higher education (greater than middle school)	1006 (32.0)	622 (41.4)	384 (23.4)	
Physical activity* (METS) median (25th–75th centile)	577.5 (82.5–1680)	773 (264–2766)	355 (0–1188)	<0.001
Past smoker†, No (%)	319 (10.1)	300 (20.0)	19 (1.2)	<0.001
Body mass index, mean (SD)	25.6 (5.5)	24.3 (4.7)	26.7 (5.8)	<0.001
Overweight/obesity‡, No (%)	2113 (67.4)	893 (59.5)	1220 (74.6)	<0.001
Waist circumference, mean (SD)	89.2 (12.5)	91.0 (12.8)	87.7 (12.1)	<0.001
Central obesity§, No (%)	2064 (65.7)	835 (55.6)	1229 (74.9)	<0.001
Waist:hip ratio, median (25th–75th centile)	0.9 (0.8–0.9)	0.96 (0.90–1.0)	0.86 (0.81–0.91)	<0.001
Glucose fasting (mmol/l), mean (SD)	6.4 (2.8)	6.3 (2.7)	6.6 (2.9)	0.005
Serum cholesterol (mmol/l), mean (SD)	4.87 (1.02)	4.76 (0.95)	4.96 (1.07)	<0.001
LDL (mmol/l), mean (SD)	2.99 (0.80)	2.94 (0.75)	3.04 (0.83)	0.003
HDL (mmol/l), mean (SD)	1.04 (0.26)	1.00 (0.22)	1.12 (0.28)	<0.001
Total lipids (g/l), mean (SD)	8.4 (1.4)	8.4 (1.5)	8.4 (1.4)	0.554
Triglycerides (mmol/l), mean (SD)	1.80 (1.08)	1.87 (1.19)	1.73 (0.96)	0.001
Cholesterol:HDL ratio, mean (SD)	4.90 (1.39)	5.18 (1.4)	4.6 (1.35)	0.007
Hypertension¶, No (%)	1341 (44.0)	501 (34.8)	840 (52.3)	<0.001
Diabetes**, No (%)	627 (21.1)	267 (19.4)	360 (23.8)	0.004
Hyperlipidaemia††, No (%)	1007 (34.9)	420 (30.5)	587 (39.0)	<0.001
High LDL‡‡, No (%)	868 (30.1)	375 (27.3)	493 (32.7)	0.001
Low HDL§§, No (%)	2087 (72.4)	929 (67.5)	1158 (76.9)	<0.001
Hypertrigyceridaemia¶¶, No (%)	1229 (42.6)	595 (43.2)	634 (42.1)	0.535
Metabolic syndrome***, No (%)	1327 (42.2)	487 (32.4)	840 (51.2)	<0.001

*Physical activity MET (metabolic equivalent) score was defined as: total MET−minutes/week  =  walk (METs−min×days) + moderate (METs×min×days) + vigorous (METs×min×days).

†Past smoker defined as those who smoked ⩾100 cigarettes in their lifetime.

‡Overweight/obesity defined as body mass index ⩾23 kg/m^2^.

§Central obesity defined as having a waist circumference of ⩾80 cm in women and ⩾90 cm in men.

¶Hypertension defined as mean systolic blood pressure ⩾140 mm Hg or mean diastolic blood pressure ⩾90 mm Hg.

**Diabetes defined as patients taking antihypertensive drugs, or having fasting blood sugar ⩾7.0 mmol/l.

††Hyperlipidaemia defined as fasting serum cholesterol of ⩾5.18 mmol/l.

‡‡High LDL defined as LDL ⩾3.37 mmol/l.

§§Low HDL defined as HDL <1.04 mmol/l in men or <1.30 mmol/l in women.

¶¶Hypertriglyceridaemia defined as serum triglyceride >1.70 mmol/l.

***Metabolic syndrome defined as the concomitant presence of at least three of the following: hypertension, high triglycerides (>1.70 mmol/l); or low HDL cholesterol (<1.04 mmol/l in men or <1.30 mmol/l in women), impaired glucose tolerance (fasting glucose ⩾6.1 mmol/l), or central obesity.

HDL, high-density lipoprotein; LDL, low-density lipoprotein.

Women were less likely than men to have had a previous diagnosis by a doctor of heart attack (3% versus 4.3%, p = 0.07), and significantly less likely to have Q waves on ECG (1.3% vs 3.1%, p = 0.001). In contrast, women were about twice as likely to return a positive response to the Rose angina questionnaire, have ischaemic changes on ECG (29.4% vs 15.6%, p<0.001), and major ECG abnormalities (table 2).

**Table 2:** Prevalence of coronary artery disease in urban Pakistan

Variables	All (n = 3143) No (%) (95% CI)	Men (n = 1503) No (%) (95% CI)	Women (n = 1640) No (%) (95% CI)	p Value
Ischaemia on ECG*				
Q-wave changes†	68 (2.2) (1.5 to 2.7)	46 (3.1) (1.9 to 4.1)	22 (1.3) (0.7 to 1.9)	0.001
Past history of heart attack diagnosed by doctor	113 (3.6) (2.5 to 4.6)	64 (4.3) (2.8 to 5.6)	49 (3.0) (1.9 to 4.0)	0.068
Past history of heart attack or Q-wave changes diagnosed by doctor	158 (5.0) (3.8 to 6.1)	97 (6.1) (4.1 to 8.0)	66 (4.0) (3.0 to 5.0)	0.009
Positive Rose angina questionnaire	398 (12.7) (10.8 to 14.5)	134 (8.9) (7.3 to 10.5)	264 (16.0) (13.4 to 18.7)	<0.001
Major ECG abnormalities‡	502 (16.0) (13.1 to 18.7)	158 (10.5) (8.1 to 12.8)	344 (21.0) (16.9 to 25.0)	<0.001

*Ischaemia on ECG: Minnesota codes 1-3 or 4-1 or 4-2 or 4-3 or 5-1 or 5-2 or 5-3 or 7-1.

†Q-wave changes: Minnesota codes 1-1 to 1-2.

‡Major ECG abnormalities: Minnesota codes: 4-1 or 4-2 or 5-1 or 5-2 or 6-1 or 6-2 or 7-1 or 7-2 or 8-1 or 8-3.

We then compared CVD risk factors in those with definite CAD (ie, those with Q waves on ECG or previous diagnosis by a doctor of heart attack), with those with no evidence of CAD on ECG and a negative response to the Rose angina questionnaire by gender. Age and hypertension, were positively and independently related to definite CAD in both genders, and diabetes in men, and waist to hip ratio in women (table 3). The amount of variance explained by the model was similar when the metabolic syndrome replaced the last three risk factors. The multivariate odds ratio (OR) for the association between the presence of the metabolic syndrome and definite CAD was equivalent in men and women (OR = 2.04 (95% CI 1.38 to 3.03) and OR = 2.08 (95% CI 1.13 to 3.829), respectively). Smoking was related to definite CAD in men but not women, the latter probably due to the paucity of ever smokers. Interestingly, none of the lipid measures were independently related to definite CAD.

**Table 3:** Factors associated with definite coronary artery disease (CAD)* in men and women

Variables	Men				Women			
	All	With CAD	Unadjusted odds ratio (95% CI)	Adjusted odds ratio (95% CI)	All	With CAD	Unadjusted odds ratio (95% CI)	Adjusted odds ratio (95% CI)
Subjects, No (%)	1211	90 (7.4)			1011	63 (6.2)		
Age (years), mean (SD)	51.3 (10.2)	56.6 (10.4)	1.05 (1.03 to 1.06)	1.03 (1.01 to 1.05)	50.9 (10.3)	56.5 (10.6)	1.02 (1.01 to 1.03)	1.03 (1.01 to 1.04)
Past smoker†, No (%)	300 (94.0)	60.0 (20.0)	3.13 (2.04 to 4.77)	2.64 (1.72 to 4.06)	15 (1.5)	1 (1.6)	0.91 (0.35 to 2.36)	NS§
Waist to hip ratio, mean (SD)	0.94 (0.08)	0.97 (0.07)	1.51 (1.19 to 1.91)	NS	0.86 (0.07)	0.90 (0.08)	1.87 (1.41 to 2.48)	1.70 (1.28 to 2.26)
Body mass index, mean (SD)	24.2 (4.7)	25.5 (4.8)	1.05 (1.01 to 1.10)	NS	26.5 (5.7)	26.3 (4.6)	0.99 (0.96 to 1.02)	NS
Hypertriglyceridaemia, No (%)	475 (39.2)	43 (47.8)	1.45 (0.95 to 2.23)	NS	387 (38.2)	29 (46.0)	1.40 (0.77 to 2.54)	NS
Low LDL, No (%)	443 (36.6)	28 (31.1)	0.76 (0.50 to 1.17)	NS	351 (34.7)	18 (28.6)	0.73 (0.50 to 1.08)	NS
Low HDL, No (%)	857 (70.7)	62 (68.9)	0.90 (0.62 to 1.31)	NS	790 (78.1)	51 (81.0)	1.20 (0.66 to 2.18)	NS
Exercise, No (%)	385 (31.8)	19 (21.1)	0.55 (0.31 to 0.95)	NS	184 (18.2)	12 (19.1)	1.06 (0.46 to 2.41)	NS
Hypertension, No (%)	351 (29.0)	46 (51.1)	2.79 (1.73 to 4.51)	2.25 (1.33 to 3.80)	467 (46.1)	46 (73.0)	3.38 (1.87 to 6.13)	2.73 (1.61 to 4.64)
Diabetes, No (%)	207 (17.0)	30 (33.3)	2.66 (1.99 to 3.56)	2.57 (1.80 to 3.68)	227 (22.4)	26 (41.3)	2.61 (1.37 to 4.95)	NS
Metabolic syndrome‡ , No (%)	543 (44.8)	55 (61.1)	2.03 (1.45 to 2.85)	Not applicable	553 (54.7)	45 (71.4)	2.16 (1.19 to 3.90)	Not applicable

*CAD defined as presence of major Q waves on ECG or history of heart attack diagnosed by a doctor.

†Past smokers defined as those who smoked ⩾100 cigarettes in their lifetime.

‡Metabolic syndrome defined as the concomitant presence of at least three of the following: hypertension, high triglycerides (>1.70 mmol/l); or low HDL cholesterol (<1.04 mmol/l in men or <1.30 mmol/l in women), impaired glucose tolerance (fasting glucose ⩾6.1 mmol/l), or central obesity.

§NS, not significant in the multivariable model and therefore not retained in the final model.

HDL, high-density lipoprotein; LDL, low-density lipoprotein.

Similarly, we compared those with ischaemia on ECG against those without definite CAD, no major ECG abnormalities, and a negative response to the Rose angina questionnaire (table 4). We noted a similar pattern of independent associations with ischaemia on ECG as we did with definite CAD. Age and hypertension were positively and independently related to ischaemic ECG in both genders. Diabetes was only statistically significant in men, and waist circumference only in women. When the metabolic syndrome replaced all risk factors (apart from age), it too was independently associated with ischaemic ECG in men and women and explained the same amount of the model variance as the individual CVD risk factors. The multivariate OR for the association between the presence of the metabolic syndrome and ischaemic ECG was similar in men and women (OR = 1.71 (95% CI 1.39 to 2.09) and OR = 1.51 (95% CI 1.22 to 1.85), respectively).

**Table 4:** Factors associated with ischaemic ECG changes* in men and women

Variables	Men				Women			
	All	With ischaemia	Unadjusted odds ratio (95% CI)	Adjusted odds ratio (95% CI)	All	With ischaemia	Unadjusted odds ratio (95% CI)	Adjusted odds ratio (95% CI)
Subjects, No (%)	1372	251 (18.3)			1011	63 (6.2)		
Age (years), mean (SD)	51.3 (10.2)	55.2 (11.6)	1.02 (1.01 to 1.04)	1.02 (1.01 to 1.03)	50.9	53.3 (11.6)	1.02 (1.01 to 1.03)	1.01 (1.00 to 1.03)
Past smoker†, No (%)	264 (19.2)	67.0 (26.7)	1.01 (0.77 to 1.30)	1.36 (0.97 to 1.89)	12 (0.84)	06 (1.2)	1.95 (0.51 to 7.44)	NS§
Waist to hip ratio, mean (SD)	0.94 (0.08)	0.96 (0.07)	0.95 (0.85 to 1.05)	NS	0.86 (0.07)	0.87 (0.07)	1.28 (1.11 to 1.47)	1.19 (1.02 to 1.38)
Body mass index, mean (SD)	24.3 (4.7)	24.9 (4.6)	1.03 (1.01 to 1.04)	NS	26.5 (5.7)	26.8 (5.5)	1.01 (1.00 to 1.01)	NS
Hypertriglyceridaemia, No (%)	546 (39.8)	114 (45.4)	1.22 1.02 to 1.47)	NS	562 (39.1)	204 (41.8)	1.18 (0.91 to 1.54)	NS
Low LDL, No (%)	491 (35.8)	76 (30.3)	0.82 (0.71 to 0.94)	NS	491 (34.2)	158 (32.4)	0.88 (0.75 to 1.03)	NS
Low HDL, No (%)	963 (70.2)	168 (66.9)	1.06 (0.90 to 1.24)	NS	1127 (78.5)	388 (79.5)	1.09 (0.83 to 1.44)	NS
Physical activity	431 (31.4)	65 (25.9)	0.71 (0.59 to 0.86)	NS	255 (17.8)	83 (17.0)	0.92 (0.67 to 1.26)	NS
Hypertension	443 (32.3)	138 (54.9)	2.71 (2.35 to 3.12)	2.81 (2.12 to 3.72)	722 (50.3)	301 (61.7)	2.01 (1.53 to 2.64)	1.82 (1.36 to 2.44)
Diabetes	238 (17.5)	61 (24.3)	1.38 (1.15 to 1.65)	1.50 (1.11 to 2.02)	314 (21.8)	113 (23.1)	1.12 (0.87 to 1.43)	NS
‡Metabolic syndrome	630 (45.9)	142 (56.6)	1.74 (1.49 to 2.01)	Not applicable	822 (57.2)	314 (64.3)	1.56 (1.26 to 1.93)	Not applicable

*Major ECG abnormalities defined as the following codes on ECG: Minnesota codes 4-1 or 4-2 or 5-1 or 5-2 or 6-1 or 6-2 or 7-1 or 7-2 or 8-1 or 8-3.

†Past smokers defined as those who smoked ⩾100 cigarettes in their lifetime.

‡Metabolic syndrome defined as the concomitant presence of at least three of the following: hypertension, high triglycerides (>1.70 mmol/l); or low HDL cholesterol (<1.04 mmol/l in men or <1.30 mmol/l in women), impaired glucose tolerance (fasting glucose ⩾6.1 mmol/l), or central obesity.

§NS, not significant in the multivariable model and therefore not retained in the final model.

HDL, high-density lipoprotein; LDL, low-density lipoprotein.

A similar exercise was performed for major ECG abnormalities as the comparator. As before, the metabolic syndrome accounted for similar model variance as individual risk factors, was an independent predictor in both genders, and had an equivalent impact in men (OR = 1.22 (95% CI 1.01 to 1.49)) and women (OR = 1.21 (95% CI 1.02 to 1.44)).

A gender comparison showed that the greater degree of definite CAD in men could be accounted for by exposure to smoking, which overwhelmed the contribution made by the metabolic syndrome (univariate OR in women compared with men OR = 0.82 (95% CI 0.57 to 1.18); multivariate OR = 0.97 (95% CI 0.69 to 1.36) (table 5). In contrast, the greater prevalence of ischaemic ECG and of major ECG changes in women compared with men could not be accounted for the by the greater prevalence of the metabolic syndrome, or by its components, in women (adjusted OR = 2.29 and 2.57, respectively) (table 5).

**Table 5:** Association of gender with ischaemic heart disease

	CAD		Ischaemia on ECG		Major ECG abnormalities	
	Unadjusted odds ratio (95% CI)	Adjusted odds ratio (95% CI)	Unadjusted odds ratio (95% CI)	Adjusted odds ratio (95% CI)	Unadjusted odds ratio (95% CI)	Adjusted odds ratio (95% CI)
Women	0.82 (0.57 to 1.18)	0.97 (0.69 to 1.36)	2.29 (1.91 to 2.76)	2.43 (1.95 to 3.02)	2.57 (2.04 to 3.24)	2.78 (2.13 to 3.64)
Age (years)	1.04 (1.02 to 1.06)	1.04 (1.02 to 1.05)	1.02 (1.01 to 1.04)	1.02 (1.01 to 1.04)	1.03 (1.02 to 1.04)	1.03 (1.01 to 1.04)
Past smoker*	2.82 (1.81 to 4.41)	2.33 (1.50 to 3.61)	1.00 (0.77 to 1.30)	1.46 (1.10 to 1.95)	0.99 (0.66 to 1.48)	1.49 (0.97 to 2.28)
Metabolic syndrome†	2.03 (1.44 to 2.85)	2.05 (1.43 to 2.94)	1.73 (1.49 to 2.01)	1.57 (1.35 to 1.84)	1.54 (1.28 to 1.86)	1.37 (1.14 to 1.64)

*Past smokers defined a those who smoked ⩾100 cigarettes in their lifetime.

†Metabolic syndrome defined as the concomitant presence of at least three of the following: hypertension, high triglycerides (>1.70 mmol/l); or low HDL cholesterol (<1.04 mmol/l in men or <1.30 mmol/l in women), impaired glucose tolerance (fasting glucose ⩾6.1 mmol/l), or central obesity.

## DISCUSSION

As far as we know, this is the first report of a large population-based study of gender differences in the prevalence and determinants of CAD in adults from an urban setting in the Indo-Pakistan subcontinent using doctors’ reports and electrocardiographic criteria. The prevalence of CAD, whichever definition was used, and especially the non-Q wave major ECG changes, was remarkably higher in this urban population than in populations of European origin,^13^ and rural populations in the Indo-Pakistan subcontinent.^8^

We confirm that definite CAD, based on a doctor’s report and Q waves on ECG, was twice as prevalent in men as in women. However, unlike populations of European origin, we not only show that ischaemic or major ECG changes were highly prevalent in our population, but that the prevalence was twice as high in women as in men. Others indicate either similar or only a modestly increased prevalence of such ECG changes.^14^ ^20^

The greater prevalence of CAD in the Indo-Pakistan population is likely to be due to a greater susceptibility to the metabolic syndrome; around a third to a half of these middle-aged men and women, respectively, were classified as positive for the metabolic syndrome using current criteria. The greater prevalence of definite CAD in men is largely due to the effect of smoking exposure, which owing to its substantially higher prevalence in men, overwhelms the impact of the greater prevalence of the metabolic syndrome in women.

Previous studies in the Indo-Pakistan subcontinent have generally reported combined definite and probable CAD prevalence, and thus not demonstrated marked gender differences^7^ However, in a study from South India, the prevalence of T-wave changes was markedly higher in women than in men (11% vs 2%), reflecting our own observations of a substantially greater prevalence of ischaemia and major changes on ECG in women.^6^ These changes are not benign, and are thought to be indicative of myocardial ischaemia, conduction abnormalities and early repolarisation reflective of structural heart disease. We show that they are strongly and equally related to the metabolic syndrome in men and women, in particular the hypertensive and dysglycaemic components. In addition, previous studies show that these changes strongly predict events and mortality, with around a twofold increased risk, even on multivariate adjustment for the presence of hypertension, diabetes, overweight and obesity, dyslipidaemia, and smoking status.^6^ ^14^ That the risk prediction is similar in Blacks and Whites suggests that these abnormalities may well be important mortality predictors in other ethnic groups such as Indo-Pakistani populations.^20^ Moreover, the identification of similar risk factors for definite CAD as well as ischaemia on ECG in our study suggests that a common underlying mechanism may be responsible for these abnormalities in this population. Thus, we suggest that ECG changes are not benign in women, and indicate that CAD may be being underdiagnosed in Indo-Pakistani women, as evident by the discrepancy between gender differences in doctor-diagnosed heart attack and ECG changes suggestive of ischaemia.

We sought to account for the greater prevalence of these changes in women by adjustment for their greater prevalence of the metabolic syndrome. However, surprisingly this made little difference to the odds ratio (2.29 to 2.43). Possible explanations for this are that we have either not properly accounted for our measured risk factors, or that there is some other unmeasured CVD risk factor, also highly prevalent in women which makes a stronger contribution. Support from the former hypothesis comes from two directions. In the UK, metabolic syndrome, and specifically insulin resistance, did not account for the greater CAD mortality in Indo-Pakistani migrants to the UK than in the general population. But an interaction was also noted between dysglycaemia and ethnicity on CAD mortality, such that diabetes increased CAD mortality by 2.78 times in Indo-Pakistanis, and only 1.46 times in Europeans.^21^ The sample was too small in that study to explore these relationships further. It is also interesting to note that among the components that constitute the metabolic syndrome, hypertension appears to be most closely related to definite CAD as well as ECG changes, particularly in women. Possibly, the duration of hypertension, in particular untreated hypertension, which has been shown to be greater in women than men, accounts for some of the greater risk of CAD in women. This is consistent with data from China which suggests that hypertension has the highest attributable risk of cardiovascular mortality in women.^22^

Our study has potential limitations. First, we did not measure the presence of atherosclerosis or infarct directly. In addition, doctor-diagnosed heart attacks were not adjudicated. However, use of ECG is a well-established and validated method for assessment of ischaemic heart disease in population-based studies.^13^ Second, ECG coding for CAD based on Minnesota coding has not been specifically validated in Indo-Pakistani populations. This requires long-term cohort data in such populations. However, correlations of these ECG changes with angiographic evidence of CAD, and with mortality have been well documented in other populations.^14^ ^23^ Third, our study was limited to the population residing in Karachi, an urban area, and therefore, may not represent the rural population of Pakistan. However, the epidemic of heart disease is likely to emerge in urban areas first, and attempts to document and stem the epidemic should begin here.^24^ Further, our findings are consistent with the recent INTERHEART study, which identified a high prevalence of CAD in this population.^25^ Our study adds to INTERHEART and other facility-based studies as our study methodology of a door to door population-based approach ensured subject selection unbiased by health seeking behaviour on the part of the subject, or by selective detailed medical evaluation on the part of the healthcare provider.^25^^–^27 Thus, we believe our rigorous study design, and the stringent, objective criteria used to define CAD maximise the generalisability, validity and reproducibility of our findings.

In conclusion, this is the first large-scale population-based study from the Indo-Pakistan subcontinent, home to one-sixth of the world’s population, on gender differences in CAD and its determinants. Our findings indicate that one in four subjects aged ⩾40 years may have underlying CAD in urban Pakistan. Increasing age, history of smoking, and the metabolic syndrome are strongly associated with CAD, and women are at greater risk than men. Doctors should be alerted to the high probability of this condition in Indo-Pakistani women, and further research conducted on why these women are at especially high risk. Concerted efforts are needed to reduce the burden of conventional risk factors for CAD, including hypertension, diabetes, dyslipidaemia, physical inactivity, tobacco use, unhealthy diets and obesity in this population, and must target both women and men.
